# Comprehensive Analysis of the Energy Harvesting Performance of a Fe-Ga Based Cantilever Harvester in Free Excitation and Base Excitation Mode

**DOI:** 10.3390/s19153412

**Published:** 2019-08-03

**Authors:** Huifang Liu, Chen Cong, Qiang Zhao, Kai Ma

**Affiliations:** School of Mechanical Engineering, Shenyang University of Technology, Shenyang 110870, China

**Keywords:** energy harvesting, Fe-Ga alloy, cantilever harvester, characteristic analysis

## Abstract

Vibration energy harvesting attempts to generate electricity through recycling the discarded vibration energy that is usually lost or dissipated, and represents an alternative to traditional batteries and may even lead to reliable self-powered autonomous electronic devices. Energy harvesting based on magnetostrictive materials, which takes advantage of the coupling characteristics of the Villari effect and the Faraday electromagnetic induction effect, is a recent research field of great interest. Aiming to develop a new type of magnetostrictive energy harvester using Fe-Ga alloy, which is suitable for harvesting the vibration energy from base excitations and free excitations, a Fe-Ga based cantilever harvester was proposed. The energy harvesting performance of the harvester prototype, including its resonance characteristics, open-circuit output voltage-frequency response and amplitude characteristic under base excitation, influence of external resistance, energy harvesting performance under free excitation, the function of pre-magnetization and so on was studied systematically and carefully by experiments. In terms of the volume power density, the harvester prototype without pre-magnetized magnet when in series with the optimal resistor load displays a value of 2.653 mW/cm^3^. The average conversion efficiency without a pre-magnetic field is about 17.7% when it is in series with a 200 Ω resistance. The energy harvesting and converting capability can therefore be improved greatly once the Fe-Ga beam is highly pre-magnetized. The prototype successfully lit up multi-LEDs and digital display tubes, which validates the sustainable power generation capacity of the fabricated prototype.

## 1. Introduction

Vibration energy harvesting attempts to generate electricity through recycling the discarded vibration energy that is usually lost or dissipated. Research results in this field can help solve many practical problems, such as powering wireless sensor networks [1,2,3], structural health monitoring [4], cardiac pacemakers [5], self-powered sensors [6], etc. In addition, it can also represent an alternative to traditional batteries and even lead to the development of reliable self-powered autonomous electronic devices. Therefore, it receives a lot of attention from academic researchers. At present, the harvested power is still far less than that of batteries, however, the latest developments in integrated circuit manufacturing, low power CMOS circuits and VLSI design have significantly reduced the power demand of commercial wireless sensors from mW to μW [7]. This enhances the feasibility of vibration energy harvesting technology for practical applications, and promotes the creation of self-powered sensor nodes and other self-powered electronic devices. Furthermore, the proposal of self-powered devices opens up new application possibilities for safety monitoring devices, structure embedded micro-sensors, and limited accessibility systems such as biomedical implants.

The most common vibration energy harvesting mechanisms are the electrostatic [8], electromagnetic [9], piezoelectric [10,11] and most recently, the magnetostrictive [12] ones. Different vibration energy harvesting mechanisms have their own respective characteristics. Piezoelectric-based and magnetostrictive-based energy harvesting technology utilizes the inherent energy conversion characteristics of smart materials. Piezoelectric-based energy harvesters convert mechanical energy into electrical energy by the direct piezoelectric effect [13]. Currently, piezoelectric-based energy harvesting technology is the most popular one because of its reasonable electro-mechanical coupling coefficient, compact configuration, no need for bulky accessories and excellent compatibility with MEMS. Zhang et al. [14] designed an arc shaped piezoelectric bistable vibration energy harvester. Zhou et al. [15] investigated a piezoelectric energy harvesting system based on the flow induced vibration of a piezoelectric composite cantilever pipe. Moreover, a method for measuring the energy harvesting efficiency was proposed. Febbo et al. [16] presented a novel design of a piezoelectric sheet rotational power scavenging system as an alternative to cantilever beams attached to a hub, which was meant to provide energy to wireless autonomous monitoring systems in low frequency environments such as wind turbines of 30 kW with rotational speeds of between 50 and 150 rpm. Guan and Liao et al. [17] proposed an innovative design of a piezoelectric rotational energy harvester which was able to generate a high output voltage at low rotation speeds with high output power over a wide rotation speed range. Santiago Orregoet et al. [18] reported a new approach to harvest ambient wind energy using an inverted piezoelectric flag fixed at the trailing edge and with the leading edge free to move. Moreover, by conducting outdoor experiments and powering a temperature sensor using the harvested ambient wind energy without storing electricity, this study may be the first example of a harvester being used in actual ambient conditions. Piezoelectric and triboelectric nanogenerators also have been proposed in the past few years to effectively harvest mechanical energy from the environment. A r-shaped hybrid nanogenerator which placed a polydimethylsiloxane layer under the aluminum electrode of polyvinylidene fluoride was proposed by Han et al. [19]. Micro/nanostructures had been fabricated on the polydimethylsiloxane surface and the aluminum electrodes of polyvinylidene fluoride aiming at enhancing the output performance. Through one cycle of electric generation, 10 light-emitting diodes could be lit up instantaneously, and a 4-bit liquid crystal display could display continuously for more than 15 s. Kim et al. [20] developed a highly flexible P(VDF-TrFE) film-based energy harvesting device on a PDMS substrate, avoiding any complex composites and patterned structures. The results showed a harvested electrical power of 6.62 mW/cm^3^ and average output voltage of 5.8 V for an active area of 4 cm^2^. Ferrari [21] from Brescia University presented a solution for battery-less power management circuits for micro-power energy converters, allowing piezoelectric energy harvesting systems to operate under continuous and intermittent conditions. However, the inherent limitations, including depolarization and aging of piezoelectric material, limit its further practical application. In addition, some piezoelectric materials have the weakness of brittleness and cannot withstand large bending strains [22]. This piezoelectric-based method which has capacitive characteristics is capable of producing high output voltage and low current. Magnetostrictive materials are a kind of metal compounds which have been gradually used in vibration energy harvesting in recent years. This method takes advantage of the coupling characteristics of the Villari effect and the Faraday electromagnetic induction effect. Vibration induces the deformation of the magnetostrictive materials, and consequently a change of magnetization in the material is produced. This change of magnetization is converted into an induced voltage or current in pick up coils surrounding the magnetostrictive materials. Magnetostrictive-based energy harvesters are capable of producing higher power density than others using lower frequency vibrations. Another advantage of magnetostrictive-based energy harvesters is that limitations such as brittleness, depolarization, aging and need for an external voltage or charge source, disappear.

At present, the most commonly used magnetostrictive material because of its merits in terms of high magneto-mechanical coupling coefficient, high energy density and excellent applicability to harsh environments is TbDyFe, which is a commercialized rare earth ferroalloy material [23]. However, it is not quite suitable for energy harvesting due to its brittleness, and difficulty to bend and miniaturize attributed to the fact TbDyFe is usually a cylinder or cuboid [24].Contrary to TbDyFe and piezoelectric stacks, Fe-Ga alloy (an iron-based material) with its advantages of high strength and ductility (tensile strength: ∼350 MPa) and better flexibility which facilitates it compatibility with flexural structures [25], may be a prospective option for harvesting vibration energy. It also has merits in terms of satisfactory robustness [26], relatively high saturation magnetostrictive coefficient (400 ppm in <100> direction) [27] and conversion efficiency (∼80%) [28], excellent thermal stability (Curie temperature > 900 K) [29], and more importantly, great machinability [30]. Therefore, Fe-Ga alloy may be more suitable for vibration harvesting using almost unlimited frequencies, with significantly improved reliability. It fills the gap between the piezoelectric stack and Terfenol-D in the field of vibration harvesting.

As mentioned, energy harvesting based on magnetostrictive materials is a recent research field of great interest. The purpose of this paper is to develop a new type of energy harvester based on Fe-Ga, which can be used for harvesting vibration energy with a wide frequency band from base excitations and free excitations. A design scheme of a Fe-Ga-based harvester prototype is presented in this paper. It is a composite cantilever beam composed of a metal base layer and a Fe-Ga alloy layer, surrounded by a pickup coil. The whole structure is capable of harvesting base excitations with low or high frequency contents, and free excitations with low frequency. A general structure of the energy harvesting and storage circuit was discussed, including a voltage quadruple rectifier circuit, a supercapacitor and an intelligent voltage regulator. Through analyzing the modeling process of a distributed-parameter model, the mechanical-magnetic-electrical fields’ coupling mechanism was studied. Energy harvesting performance of the harvester prototype was studied systematically and carefully by experiments. The sustainable power generation capacity of the fabricated prototype was validated through keeping lit up multi-LEDs and digital display tubes.

## 2. Prototype

**Figure 1:**
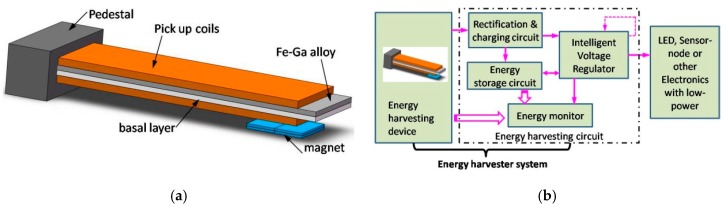
Structure of the proposed Fe-Ga-based cantilever vibration harvester: (**a**) harvesting device; (**b**) energy harvesting and storage circuit.

The Fe-Ga-based cantilever vibration energy harvester consists of two parts: a harvesting device comprised of magnetostrictive material (a Fe-Ga layer) bonded on a basal layer wound by pick up coils, as shown in Figure 1a; and an energy harvesting and storage circuit. On the whole, the proposed vibration harvesting device is a composite beam structure. The magnetically easy axis of Fe-Ga alloy is in the longitudinal direction. Fe-Ga alloy is stress annealed under a compressive stress to provide built-in uniaxial anisotropy so that magnetic flux variation occurs under compressive as well as tensile stresses. The pick up coils layer is solidified by using 3M DP100 two-component epoxy adhesive glue. When we study the influence of the pre-magnetization field on the energy harvesting performance experimentally, it can attach a permanent magnet near the composite beam in order to generate a pre-magnetization field. The harvesting device can receive base excitation with any frequency or free excitation at lower frequency, and a voltage is generated on the pick up coils as follows: when a vibration is applied to the beam through base excitation or free excitation, the structure bends like a cantilever, i.e., bending strain and stress are generated in the beam. Vibration strain caused by bending will result a change of the magnetic flux in the Fe-Ga layer through the Villari effect [31]. By vibrating the device dynamically or cyclically, the time variation of fluxes caused by periodic bending deformation generates a voltage on the pick up coils according to Faraday’s law of induction [32]. Then, the induced voltage is harvested and stored as electric energy by the whole energy harvesting and storage circuit, which supplies power to electronic components according to their requirements.

The energy harvesting and management unit consists of a rectification and charging circuit, an energy storage circuit, an intelligent voltage regulator and an energy monitor. The intelligent regulator resembles an intelligent voltage valve. It can not only adjust the voltage from the harvester to meet the required voltage range, but also can shut down automatically when the energy storage cell cannot withstand consumption. The function of the energy monitor is to track the available energy from the environment, as well as the state of the energy storage cell. These data will be input into the power management algorithm for learning the energy environment so as to better manage the energy harvesting and storage circuit.

## 3. Analysis of the Energy Harvesting and Conversion Mechanism

There are three coupling domains when energy is harvested: the mechanical domain, magnetic domain and electric domain. Mechanical energy, i.e., vibration, force or motion, first acts on the beam, which leads to the Fe-Ga beam bending and produces strain or stress in the beam in the mechanical domain. Then, the stress or strain induces the a Villari effect the Fe-Ga alloy, which leads to a magnetic flux. In this stage, the mechanical domain is coupled with the magnetic domain, and mechanical energy is transformed into magnetic energy. Any variation of magnetic flux results in a Faraday electromagnetic induction effect, and correspondingly an induced electromotive force is generated in the pick up coils. The magnetic domain is coupled with the electric domain, and thus, magnetic energy is converted into electric energy.

This means that the Fe-Ga harvester harvests mechanical energy and converts it into electrical energy mainly in three stages: mechanical bending occurring in the beam in the mechanical domain, flux changing accompanied with bending, and a flux producing electrical energy. Stress (strain) and magnetization (flux density) are the linking variables between these three stages, respectively. Therefore, in this section, the mathematical mechanism of energy harvesting and conversion of Fe-Ga energy harvester is analyzed by the distributed-parameter modeling method considering the above three domains.

### 3.1. Mechanical Bending Process of Fe-Ga

**Figure 2:**
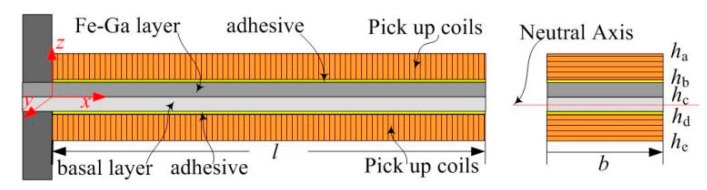
Simplified mechanical schematic diagram of the harvester; the inset shows a diagram of the cross section.

As shown in Figure 2, the proposed Fe-Ga alloy vibration harvester is approximately equivalent to a 4-layer composite beam consisting from the bottom to the top of a pick up coils layer, a basal layer, a Fe-Ga alloy layer and a pick up coils layer. The elastic modulus of the 3M DP100 two-component epoxy adhesive glue used is quite small compared with that of basal layer or Fe-Ga layer, thus we can neglect its thickness and action on bending. The boundary positions of every layer relative to the neutral axis are expressed as *h_a_*, *h_b_*, *h_c_*, *h_d_*, and *h_e_*. The equivalent length and width of the beam are *l* and *b*, respectively. The longitudinal displacement *w*(*x*, *t*) (in the *z*-axis direction) under free excitation is determined by the Euler-Bernoulli beam theory [33]. Therefore, the motion governing equation for free vibration of the composite beam in the range of 0≤x≤l is given as [34]:

(1) $$C_{b}\frac{\partial w\left(x,t\right)}{\partial t}+m\frac{\partial^{2}w\left(x,t\right)}{\partial t^{2}}=\frac{\partial^{2}M_{b}\left(x,t\right)}{\partial x^{2}}$$

in which, *C_b_* is the equivalent damping coefficient. *M_b_*(*x*, *t*) is the internal bending moment in the *z*-axis, and *m* is the mass per unit length of the beam, which are respectively expressed as:

(2) $$M_{b}\left(x,t\right)=-EI\frac{\partial^{2}w\left(x,t\right)}{\partial x^{2}}=-\frac{b}{3}\left[E_{s}\left(h_{c}^{3}-h_{d}^{3}\right)+E_{G}\left(h_{b}^{3}-h_{c}^{3}\right)+E_{c}\left(h_{a}^{3}-h_{b}^{3}\right)+E_{c}\left(h_{d}^{3}-h_{e}^{3}\right)\right]\frac{\partial^{2}w\left(x,t\right)}{\partial x^{2}}$$

(3) $$m=b\left(\rho_{\mathrm{st}}h_{\mathrm{st}}+\rho_{\mathrm{MsM}}h_{\mathrm{MsM}}\right)$$

In Equation (2), *E* and *I* are the equivalent elastic modulus and the moment of inertia, and *EI* is the total equivalent flexural rigidity of the composite beam. The total equivalent elastic modulus is expressed as a multi single elastic modulus of each layer; *E*_s_, *E_G_* and *E*_c_ are the elastic moduli of the basal layer, Fe-Ga layer and pick up coils layer, respectively. In Equation (3), the symbols 𝜌 and *h* represent the mass density and thickness, respectively. Subscripts ‘st’ and ‘MsM’ represent the basal layer and Fe-Ga alloy layer, respectively.

Boundary conditions for the mechanical motion of beam are that the displacement and velocity at the fixed end are zero; and, the acceleration and jerk at the free end are all zero. The corresponding expressions of boundary conditions are [34]:

at the clamping end:

(4a) $$w\left(0,t\right)={\frac{\partial w\left(0,t\right)}{\partial x}|}_{x=0}=0$$

at the free end:

(4b) $${\frac{\partial^{2}w\left(x,t\right)}{\partial x^{2}}|}_{x=l}={\frac{\partial^{3}w\left(x,t\right)}{\partial x^{3}}|}_{x=l}=0$$

The solution of Equation (1) can be gained using the variable-separation method and can be expressed by a normal mode shape function:

(5) w(x,t)=S(x)T(t)

here, *S*(*x*) is the normal mode shape function; *T*(*t*) is the generalized coordinate function.

The displacement normal mode shape function *S*(*x*) satisfies the following equation relations under the assumption of Euler-Bernoulli beam theory and boundary conditions:

(6) $$S^{\left(4\right)}\left(x\right)-\frac{m\omega^{2}}{EI}S\left(x\right)=0$$

The general solution of the displacement normal mode shape function is expressed by the following equation [35]:

(7) $$\begin{array}{cc} S\left(x\right) & =A_{s1}\cosh\left(\sqrt{\omega }{\left(\frac{m}{EI}\right)}^{1/4}x\right)+A_{s2}\sinh\left(\sqrt{\omega }{\left(\frac{m}{EI}\right)}^{1/4}x\right)+A_{s3}\cos\left(\sqrt{\omega }{\left(\frac{m}{EI}\right)}^{1/4}x\right)+A_{s4}\sin\left(\sqrt{\omega }{\left(\frac{m}{EI}\right)}^{1/4}x\right)\\ & =A_{s1}\cosh\left(\lambda \frac{x}{l}\right)+A_{s2}\sinh\left(\lambda \frac{x}{l}\right)+A_{s3}\cos\left(\lambda \frac{x}{l}\right)+A_{s4}\sin\left(\lambda \frac{x}{l}\right) \end{array}$$

where we set (λl)4=mω2EI; The constant coefficients *A_si_*, (*i* = 1, 2, 3, 4) are determined by the boundary conditions in the equation:

(8) $$\left\{ \begin{array}{l} A_{s1}=-A_{s3}\\ A_{s2}=-A_{s4} \end{array}\right.\left[\begin{array}{cc} \cosh\lambda +\cos\lambda & \sinh\lambda +\sin\lambda \\ \sinh\lambda +\sin\lambda & \cosh\lambda +\cos\lambda \end{array}\right]\left[\begin{array}{c} A_{s1}\\ A_{s2} \end{array}\right]=\left[\begin{array}{c} 0\\ 0 \end{array}\right]$$

For the nontrivial solution of λ, setting the determinant to be zero results in the following relation [34]:

(9) coshλcosλ+1=0

By solving the above transcendental equation with the numerical analysis method, λ and *A*_s*i*_ are determined, and then displacement normal mode shape of the beam can be further obtained by Equation (7).

The normal mode shape function solution can be rewritten as:

(10) $$S\left(x\right)=\cosh\lambda \frac{x}{l}-\cos\lambda \frac{x}{l}-A_{s}\left(\sinh\lambda \frac{x}{l}-\sin\lambda \frac{x}{l}\right)$$

where, $A_{s}=A_{s2}/A_{s1}=\left(\sin\lambda -\sinh\lambda \right)/\left(\cosh\lambda +\cos\lambda \right)$.

For a pure bending beam, the stress formula is:

(11) $$\sigma =\frac{M_{\mathrm{MsM}}\left(x,t\right)w\left(x,t\right)}{I}$$

where, *M*_MsM_(*x*, *t*) is the bending moment in *z*-axis of Fe-Ga layer. Replacing parameter *E* of Equation (2) by *E_G_*, and substituting the equation into Equation (11), we get the stress in Fe-Ga layer which is expressed by longitudinal displacement:

(12) $$\sigma \left(x,z,t\right)=-zE_{G}\frac{\partial^{2}w\left(x,t\right)}{\partial x^{2}}=-zE_{G}S^{\prime \prime }\left(x\right)T\left(t\right)$$

### 3.2. The Process of Flux Change Caused by Bending

Magnetic flux appears in the beam with its bending, which indicates that there is coupling between the mechanical domain and the magnetic domain. Accordingly, one needs to make clear the magnitude of magnetization and magnetic flux density caused by stress. There have been several models developed for describing the mathematical relationship between stress and magnetization of magnetostrictive materials, including linear piezomagnetic equation [36], finite element uncoupled and coupled model [37,38], and the distributed-parameter dynamic coupled model [39]. In the harvester proposed in this paper there is a pre-magnetic magnet near the Fe-Ga beam, therefore, it is necessary to consider the effect of a pre-magnetization field on the Villari effect. We selected the J-A magneto-mechanical coupling theory [40] to describe the variation of magnetic flux in Fe-Ga with bending stress.

The J-A magneto-mechanical coupling theory holds that non-magnetic inclusions, grain boundaries, internal stress and other constraints hinder the magnetization process resulting from the domain wall substitution, which further leads to a hysteresis. In addition, the total magnetization *M* is divided into two parts: a reversible component *M*_rev_ and an irreversible component *M*_irr_.

Here, the reversible magnetization is linear with the difference between non-hysteresis magnetization and irreversible magnetization [41,42]:

(13) $$M_{\mathrm{rev}}\left(x,z,t\right)=\kappa \left[M_{\mathrm{an}}\left(x,z,t\right)-M_{\mathrm{irr}}\left(x,z,t\right)\right]$$

in which, the parameter *M*_an_ represents the anhysteretic magnetization, κ is the irreversible loss coefficient.

Accordingly, the total magnetization can be further expressed as a linear superposition of non-hysteresis magnetization and irreversible magnetization:

(14) $$M\left(x,z,t\right)=M_{\mathrm{rev}}\left(x,z,t\right)+M_{\mathrm{irr}}\left(x,z,t\right)=\kappa M_{\mathrm{an}}\left(x,z,t\right)+\left(1-\kappa \right)M_{\mathrm{irr}}\left(x,z,t\right)$$

For the anhysteretic magneto-mechanical behavior, we constructed a mathematical expression using the Langevin function [43] with the consideration of the effective intensity of magnetic field *H*_e_. The relationship between anhysteretic magnetization and the effective intensity of magnetic field is not linear, but a relationship involving hyperbolic cotangent function and derivative, which is expressed as [41,42]:

(15) $$M_{\mathrm{an}}\left(x,z,t\right)=M_{s}\left[\coth\left(\frac{H_{e}\left(x\right)}{\gamma }\right)-\frac{\gamma }{H_{e}\left(x\right)}\right]$$

In Equation (15), γ is the coefficient of anhysteretic magnetization; *M*_s_ is the saturation magnetization.

The actual effective magnetic field in Fe-Ga alloy includes not only the pre-magnetic field *H*(*x*), but also another magnetic field generated by stress, $\frac{9\lambda_{s}\sigma \left(x,z,t\right)}{2\mu_{0}M_{s}^{2}}M\left(x,z,t\right)$, which is due to the bidirectional energy conversion characteristics of the magnetostrictive material. In addition to this, the spontaneous magnetization effect [44] also generates a magnetic field called the internal magnetic field of the external molecule, which is with the magnitude of τM(x,y,t). Correspondingly, the effective magnetic field intensity and anhysteretic magnetization in Fe-Ga become:

(16) $$H_{e}\left(x\right)=H\left(x\right)+\tau M\left(x,z,t\right)+\frac{9\lambda_{s}\sigma \left(x,z,t\right)}{2\mu_{0}M_{s}^{2}}M\left(x,z,t\right)$$

(17) $$M_{\mathrm{an}}\left(x,z,t\right)=M_{s}\left[\coth\left(\frac{H\left(x\right)+(\tau +\frac{9\lambda_{s}\sigma \left(x,z,t\right)}{2\mu_{0}M_{s}^{2}})M\left(x,z,t\right)}{\gamma }\right)-\frac{\gamma }{H\left(x\right)+(\tau +\frac{9\lambda_{s}\sigma \left(x,z,t\right)}{2\mu_{0}M_{s}^{2}})M\left(x,z,t\right)}\right]$$

In Equations (16) and (17), λ_s_ is the saturation magnetostriction coefficient; μ_0_ is vacuum permeability; *τ* is the magnetic coefficient of external molecule.

Another component of the total magnetization during the Villari effect is the irreversible magnetization whose relationship with the stress is expressed as [41,42]:

(18) $$M_{\mathrm{irr}}\left(x,z,t\right)=M_{\mathrm{an}}\left(x,z,t\right)-\frac{E_{MsM}\xi }{\sigma \left(x,z,t\right)}\frac{dM_{\mathrm{irr}}}{d\sigma }$$

where, *ξ* is the energy coupling parameter per unit volume of Fe-Ga alloy.

Equations (14), (17) and (18) describe the action of the Villari effect excited by vibration and pre-magnetic field from the mathematical perspective. The pre-magnetization field generated by a permanent magnet is not absolutely uniform along the longitudinal direction in the Fe-Ga beam. It may be more simple to adopt the finite element numerical analysis method to analyze and determine the intensity.

The above content clearly describes the relationship between magnetization and stress, however, we need to further establish the mathematical relationship between magnetic flux density and magnetization in order to determine the variation of magnetic flux upon bending the Fe-Ga beam. We derive the relationship between magnetic flux density and total magnetization through Hopkinson’s law and the magneto motive force. Because of the unclosed total magnetic circuit in the harvester, the magnetic distribution in air part is uneven in an infinite spatial space. Consequently, it is an extremely difficult and time-consuming computation to get an accurate distribution of magnetic flux. Thus, for simplicity, we regard the beam as an elongated spheroid and adopt a demagnetizing field and a demagnetizing factor to evaluate the magnetic flux density approximately. The relationship between magnetic flux density and magnetization is then approximately expressed as [45]:

(19) $$B\left(x,z,t\right)=\mu_{0}H_{d}=\mu_{0}N_{d}M\left(x,z,t\right)$$

(20) $$N_{d}=\left(\frac{1}{1-{\left(b/l\right)}^{2}}-1\right)\left[\frac{1}{2\sqrt{1-{\left(b/l\right)}^{2}}}\log\frac{1+\sqrt{1-{\left(b/l\right)}^{2}}}{1-\sqrt{1-{\left(b/l\right)}^{2}}}-1\right]$$

in Equation (19), *H*_d_ and *N*_d_ are the demagnetizing field and demagnetizing factor, respectively.

### 3.3. Electrical Power Generation Process

#### 3.3.1. Open-Circuit Voltage Output by the Harvester

Energy flows from the magnetic domain to the electrical domain with the aid of the magnetic flux which is changing with time, and finally an induced electromotive force is generated in the pick up coil through the occurrence of the Faraday electromagnetic induction effect. According to the obtained magnetic flux density described in Equation (19), the induced voltage is obtained finally as:

(21) $$u=N\frac{\int_{0}^{l}\frac{\partial \Phi \left(x,t\right)}{\partial t}dx}{l}=N\frac{\int_{0}^{l}\left[\int_{h_{c}}^{h_{b}}\frac{\partial B\left(x,z,t\right)}{\partial t}bdz\right]dx}{l}$$

where, *N* is the turns of pick up coil.

#### 3.3.2. The Harvester Provides Electrical Power for External Pure Resistance Load

When the harvester powers an electronic component which internal electrical load is only a resistor, it is equivalent to a situation where the pick up coil is directly connected with an external pure resistance load at that moment. The output voltage from the pick up coil is AC, which also means that the circuit has a AC voltage source. Therefore, in this paper, the output power from the harvester, that is, the power consumed by the external resistance, is calculated by the analytic phasor method. If the output voltage is a standard sinusoidal periodic signal:

(22) $$u=U_{m}\sin\left(\omega t+\psi \right)$$

in which, *U_m_* is amplitude of the voltage at the both ends of pickup coil, ψ is the phase angle. When the pick up coil with a impedance *Z*_pm_ (*Z*_pm_ = *R*_p_ + *jX*_pL_) is connected with a resistive load *R*_e_, the current in the circuit is:

(23) $$i=\frac{u}{R_{e}+Z_{\mathrm{pm}}}=\frac{\left|U_{m}\right|∠\psi }{R_{e}+R_{p}+jX_{\mathrm{pL}}}$$

where, *R*_p_ and *X*_pL_ are the resistance value and inductive impedance which is equal to *L*ω = *L*2π*f*; *L* is the inductance of pick up coil, and *f* is frequency. Finally, the expression of current is derived as:

(24) $$i=\frac{\left|U_{m}\right|∠\left(\psi -\arctan\frac{X_{\mathrm{pL}}}{R_{p}+R_{e}}\right)}{\sqrt{{\left(R_{p}+R_{e}\right)}^{2}+X_{\mathrm{pL}}^{2}}}$$

In order to calculate the output electrical power, the RMS current should be determined first:

(25) $$I=\frac{U_{m}}{\sqrt{2}\sqrt{{\left(R_{p}+R_{e}\right)}^{2}+X_{\mathrm{pL}}^{2}}}$$

If the output voltage is a non-standard sinusoidal periodic signal, the formula for the RMS current mentioned above is no longer applicable. In this case, a general formula for calculating the root mean square value of current is adopted, in which, it uses the discrete voltage values in a whole period. And it can be expressed as follows:

(26) $$I=\frac{U}{\sqrt{{\left(R_{\mathrm{pL}}+R_{e}\right)}^{2}+X_{\mathrm{pL}}^{2}}}=\frac{\sqrt{\frac{1}{T_{0}}\int_{0}^{T_{0}}u^{2}dt}}{\sqrt{{\left(R_{\mathrm{pL}}+R_{e}\right)}^{2}+X_{\mathrm{pL}}^{2}}}$$

in which, *U* is the root mean square value of the output voltage, *T*_0_ is the voltage signal period which is reciprocal with frequency *f*.

Then, the output power *P*_o_ can be calculated by the following methods:

For a standard sinusoidal voltage signal:

(27a) $$P_{o}=I_{e}^{2}R_{e}=\frac{U_{m}^{2}R_{e}}{2\left[{\left(R_{p}+R_{e}\right)}^{2}+X_{\mathrm{pL}}^{2}\right]}$$

For a non-standard sinusoidal voltage signal:

(27b) $$P_{o}=I_{e}^{2}R_{e}=\frac{1}{T_{0}}\frac{R_{e}}{{\left(R_{\mathrm{pL}}+R_{e}\right)}^{2}+X_{\mathrm{pL}}^{2}}\int_{0}^{T_{0}}u^{2}dt$$

The maximum output power from the harvester can be easily obtained through finding the extremum of Equation (27a) or (27b) according to whether the voltage signal is standard sinusoidal or not. The optimal external resistance *R*_e-op_ corresponding to the maximum output power can be calculated by finding the zero derivatives of Equation (27a) or (27b). Then, the maximum power and the optimum resistance are determined as:

(28) $$P_{o-\max}=\frac{1}{T_{0}}\frac{\sqrt{R_{p}^{2}+X_{\mathrm{pL}}^{2}}}{{\left(R_{p}+\sqrt{R_{p}^{2}+X_{\mathrm{pL}}^{2}}\right)}^{2}+X_{\mathrm{pL}}^{2}}\int_{0}^{T_{0}}u^{2}dt=\frac{U^{2}\sqrt{R_{p}^{2}+X_{\mathrm{pL}}^{2}}}{2\left(R_{p}^{2}+X_{\mathrm{pL}}^{2}+R_{p}\sqrt{R_{p}^{2}+X_{\mathrm{pL}}^{2}}\right)}$$

(29) $$R_{e-\mathrm{op}}=\sqrt{R_{p}^{2}+X_{\mathrm{pL}}^{2}}$$

The power density of the Fe-Ga-based cantilever harvester adopts the form of a volumetric specific power, that is, the output power of per unit volume of effective material per unit time. Here, the volume of Fe-Ga layer is used to calculate power density. It is expressed as:

(30) $$\chi_{P}{}_{o}=\frac{P_{o}}{V_{\mathrm{MsM}}}=\frac{P_{o}}{lb_{\mathrm{MsM}}h_{\mathrm{MsM}}}$$

where, *V*_MsM_ is the volume of Fe-Ga layer, which is equal to the product of length, width and height, for the thin cuboid Fe-Ga layer.

Energy harvesting and conversion efficiency is defined as output electrical power taken out at the resistance as Joule loss divided by the work conducted on the harvester by the following equation:

(31) $$\eta =\frac{W_{o}}{W_{i}}$$

If the harvester works in a free excitation situation, which is generated by an initial excitation force *F*_0_ with initial displacement *X*_0_ on the free end of the beam, it will generate a voltage in the time range 0—*t*_f_. In this case, the energy harvesting and conversion efficiency is expressed as:

(32) $$\eta =\frac{W_{o}}{W_{i}}=\frac{\int_{0}^{t_{f}}\frac{U_{R}^{2}}{R_{e}}}{\frac{1}{2}F_{0}X_{0}}$$

in which, *U*_R_ is the voltage on the external resistor.

### 3.4. Numerical Calculation Method

**Figure 3:**
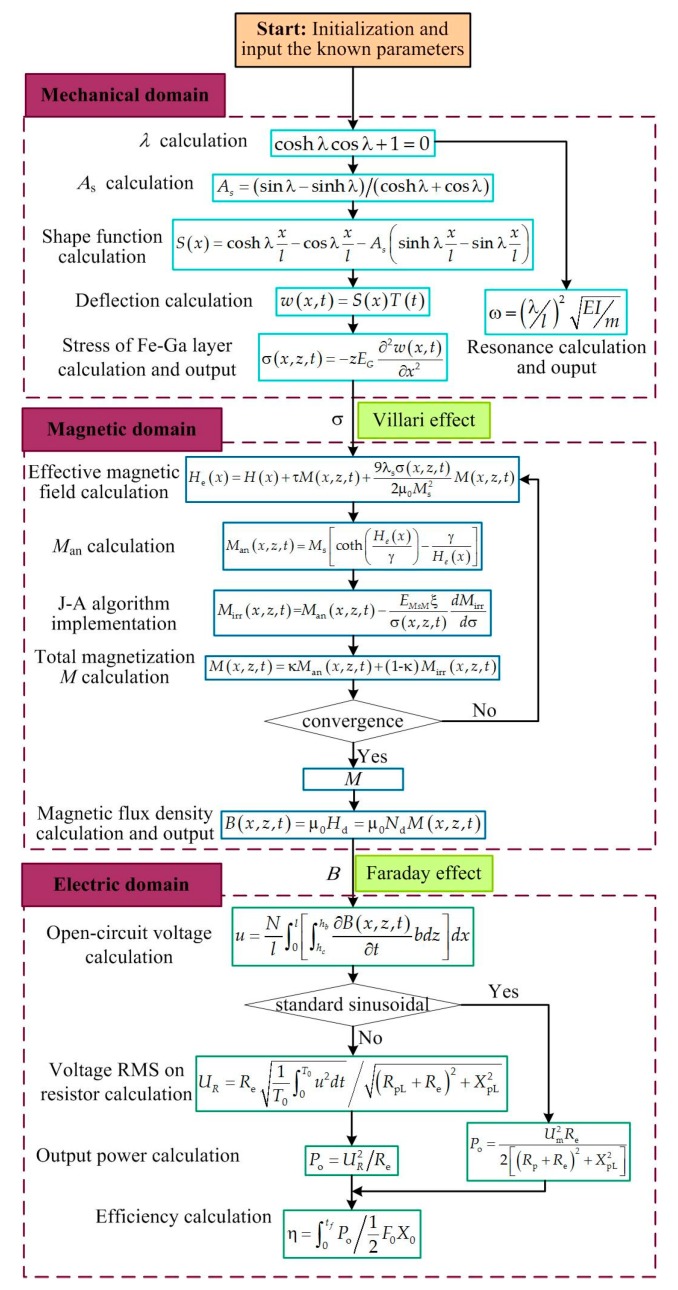
Model algorithm of the Fe-Ga energy harvester.

**Figure 4:**
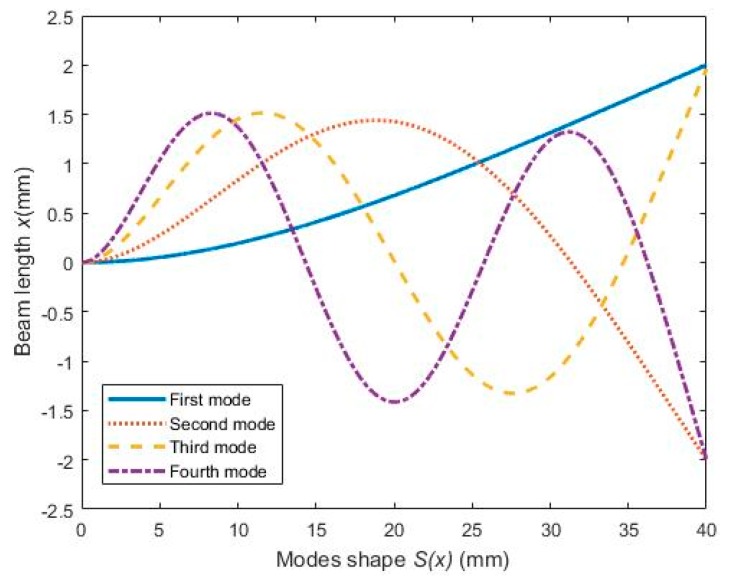
The modes shape of the beam.

The mathematical equations stated above allow one to analyze the basic phenomena occurring in a magnetostrictive vibration harvester in the case that pre-magnetization field is taken into account. It consists of three parts, which are mechanical bending vibration submodel, magneto-mechanical coupling submodel, and magneto-electric submodel. The parameter stress *σ* determined from the mechanical bending vibration submodel is the input variable of magneto-mechanical coupling submodel whose output variable is the magnetic flux density *B*; the parameter *B* links the magneto-mechanical coupling and magneto-electric submodels. In this paper, numeric iterative method is used to solve the magnetization coupled problem. For example, ordinary differential equations are solved through the midpoint method, and nonlinear equation sets are solved through the Newton iteration method. Firstly, the stress and magnetic flux density are calculated and substituted into magneto-mechanical coupling submodel and magneto-electric submodel, respectively, then the output voltage can be calculated. On this basis, the output power and energy conversion efficiency of the system are calculated using the root mean square value of voltage or current according to whether the voltage signal is a standard sinusoidal one or non-standard. Figure 3 illustrates the diagram of the calculation process that has been implemented by Matlab. It includes three segregated steps, one step for the mechanical problem, the second step for the magnetic and magnetostrictive problem, and another step for the electromagnetism problem. The system parameters involved in the numerical analysis are listed in Table 1. The first four resonance frequencies are calculated, which are 65.3, 206.5, 349.7 and 415.6 Hz, respectively. To simplify the calculations, we assume that in the resonance area, the modes can respond individually. This assumption is generally not valid [46] because the assumed modes do not reflect the actual behavior of the beam. The four consecutive modes shape are obtained and shown in Figure 4. The calculation results will be analyzed and compared with experimental results in Section 4.3.

Analyzing Equation (17), it is known that magnetization is related to the intensity of pre-magnetization field closely, correspondingly, magnetic flux density and induced voltage are all related to the pre-magnetization field too (according to Equations (19) and (20)). A pre-magnetization field is important to ensure the largest variation magnetic flux and induced voltage, but it’s not that the stronger the magnetic field, the better; maybe there is an optimal pre-magnetization state. Analyzing Equation (21), it is also known that output voltage is proportional to the time variation of magnetic flux, in other words, it is proportional to the velocity of the beam. Thus, it may conclude that the vibration frequency has an influence on the energy harvesting capability. We will further analyze how these main factors affect the induced voltage and energy conversion by experiments.

## 4. Comprehensive Analysis of Energy Harvesting Characteristics

**Figure 5:**
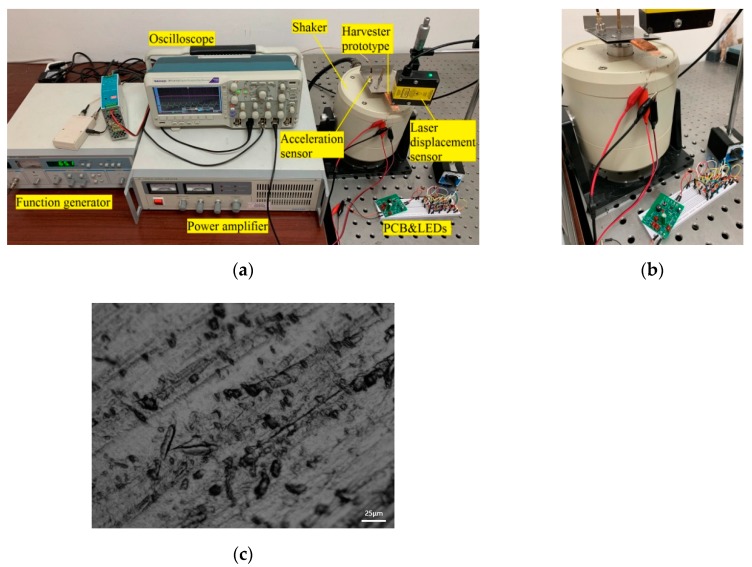
Photographs of experimental setup and harvester prototype: (**a**) Experimental setup; (**b**) a close-up of the actual fabricated harvester prototype; (**c**) SEM picture of the Fe-Ga material.

The experimental setup and close-up of the actual fabricated Fe-Ga based cantilever harvester prototype are shown in Figure 5a,b, respectively. The dimensions of the Fe-Ga layer made by NanFang Rare Earth Metal Material Co., Ltd. (Huizhou, China) are 40 mm × 15 mm × 0.5 mm, the basal layer with dimensions of 40 mm × 15 mm × 0.3 mm is made of beryllium copper, and the winding pick up coils manufactured by the TaiXingLongChang Electric Heating Element Co., Ltd. (Taixing, China) are 800 turns of 0.23 mm diameter wire. A SEM picture of the Fe-Ga material observed by a Keynes Microsystem made by Keyence Corporation (Osaka, Japan) is shown in Figure 5c. A YE1311 function generator (Sinocera, Yangzhou, China) excites a sinusoidal signal. Then the signal is sent to a Sinocera YE5873A wideband power amplifier to drive a Sinocera JZK-20 vibration shaker. The Fe-Ga based harvester prototype is screwed to the top end of a JZK-20 shaker (Sinocera, Yangzhou, China) with the aid of an auxiliary fixture. Meanwhile, a Microtrak II laser displacement sensor (MTI Instruments INC, Albany, NY, USA) measures the time-varying displacement of the harvester.

A Sinocera Piezotronics CA-YD-181 acceleration sensor with a sensitivity of 3 pc/g measures the time-varying vibration acceleration of the harvester. The signals of the laser sensor, acceleration sensor, and harvester’s output voltage are recorded by the three different channels of a DPO2014B digital phosphor oscilloscope (Tektronix, Tektronix Inc. Beaverton, OR, USA).

### 4.1. Resonance Characteristics

The Lissajous figures method [48] is used to measure the resonant frequency of the vibration harvesting device. A sinusoidal harmonic oscillation is applied on the harvesting device by a vibration shaker which excitation signal is generated by a function waveform generator. The excitation signal and vibration acceleration signal of the harvesting device are simultaneously fed into the two channels of a digital oscilloscope. Then, the signals from function waveform generator and acceleration sensor are setting as the *X*-axis input and *Y*-axis input, respectively. The excitation frequency is continuously adjusted by applying a manual sinusoidal sweep mode. Then the natural frequencies of the harvesting device can be determined through synthetically observing the Lissajous figures.

**Figure 6:**
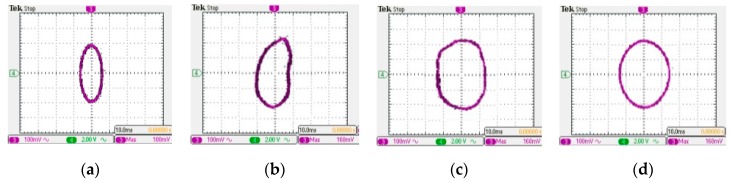
Lissajous figures for measuring resonant frequency: (**a**) 65 Hz; (**b**) 210 Hz; (**c**) 330 Hz; (**d**) 405 Hz.

Analyzing the harvesting device involved in this paper, it is noted that there is a 90° phase difference between input vibration signal and output acceleration signal. Therefore, the Lissajous figures are approximate ellipses when the system resonates; correspondingly, we can determine the resonant frequencies of the harvesting device through finding elliptical Lissajous figures. We have observed four elliptical Lissajous figures in turn below 500 Hz, as shown in Figure 6, whose corresponding frequencies are 65, 210, 330 and 405 Hz, respectively. Thus the first four resonances of the harvesting device are obtained: the fundamental natural frequency is about 65 Hz, and the second-fourth order resonant frequencies are 210, 330 and 405 Hz, respectively. Comparing with the calculation results in Section 3.4, it is found that the measured values of the first four resonant frequencies are approximately in agreement with the simulation results, and the larger difference occurs at the third and fourth resonance. This may result from inaccurate estimation of the total equivalent flexural rigidity of the composite beam. Consequently, for the vibration energy harvester prototype designed in this paper, the fundamental natural frequency is relatively low, and its better working condition may be in the low frequency vibration region. If it is hoped to be used for harvesting high frequency vibration, one may use an additional mass to tune the resonant frequency to match that of the vibration source.

### 4.2. Effect of Excitation Frequency and Amplitude on Open-Circuit Output Voltage under Base Excitation

**Figure 7:**
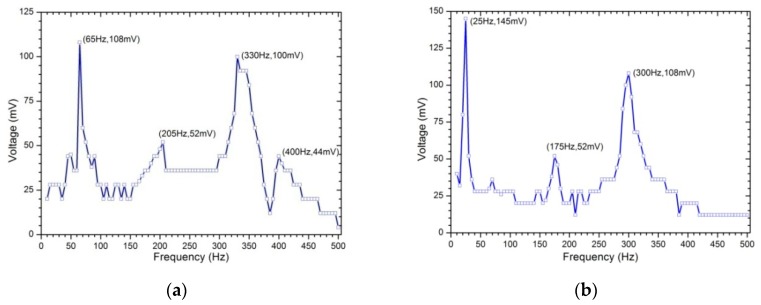
Voltage frequency-response: (**a**) without additional mass; (**b**) with 0.01 Kg mass.

Under a sinusoidal vibration with constant acceleration amplitude of 3.6 g, the output voltage-frequency response of the harvester is shown in Figure 7a. The output voltage has four resonances, which are 65, 205, 330 and 400 Hz under 500 Hz. The voltage at the first natural frequency is the largest, which has 108 mV amplitude. Under the same excitation, the voltage-frequency response of the harvester with an 0.01 Kg additional mass block at the free end is also measured in the same frequency range, as shown in Figure 7b. It can be seen that the existence of additional tip mass changes both the natural frequency and the ability to generate voltage. In particular, an additional mass significantly affects the natural frequency of the energy harvesting system, that is, it reduces the natural frequency compared with a situation with no additional mass. In this case, we observed that the harvester’s resonances occurred at 25, 175 and 300 Hz. On the other hand, an additional tip mass increases the resonant voltage. For example, the first-order resonant voltage is 108 mV without an additional mass, while it is 145 mV with an additional mass; the third-order resonant voltage is 100 mV without an additional mass, while it is 108 mV with an additional mass. In addition, the voltage generated by the harvester designed in this paper is larger at the first- and third- order natural frequencies, and the first-order resonant voltage is slightly larger than that of the third-order natural frequency. However, the voltage at the second-order natural frequency is the smallest. Therefore, in order to obtain the best harvesting performance in different vibration environments, it may adjust the natural frequency through attaching an additional mass to the tip of the beam to make its resonant frequency be consistent with the low-frequency vibration environment. The additional mass will reduce the generated voltage in theory because the voltage is proportional to the frequency according to Faraday’s law of induction, however, the advantage of using the tip mass is increasing the forcing vector, leading to larger harvested energy.

**Figure 8:**
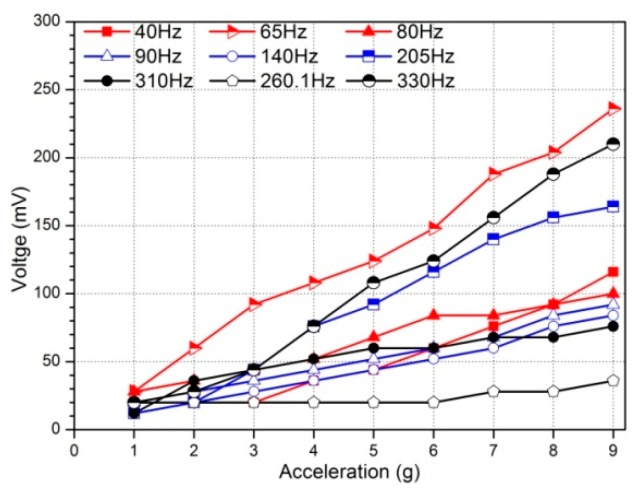
Voltage versus excitation acceleration at nine group frequencies.

A relationship between output and input of the harvester at different frequencies is obtained through experiments, that is, the relationship between voltage and acceleration amplitude, as shown in Figure 8. As can be seen from the curves, the voltage increases with acceleration. The relationship between voltage and acceleration is somewhat linear, but it is not completely linear. In the nine groups of measured frequency, there are three change voltage cycles. The voltage increases from 40 Hz to 65 Hz, decreases from 65 Hz to 140 Hz, then increases to 205 Hz, decreases to 260 Hz, and finally increases to 330 Hz. The top three lines correspond to the first, third and second resonant frequency respectively, from top to bottom, which are 65, 205 and 330 Hz. It can be seen that under a same acceleration, the output voltage is the largest at 65 Hz, that is, the first-order resonance voltage is the largest. When it works far from resonance, the sensitivity of voltage to excitation amplitude is relatively poor, that is, the voltage no longer changes generally. For example, the voltage- acceleration curve is approximately horizontal, when the harvester works at 260 Hz.

### 4.3. Power and Power Density Affected by External Resistance

**Figure 9:**
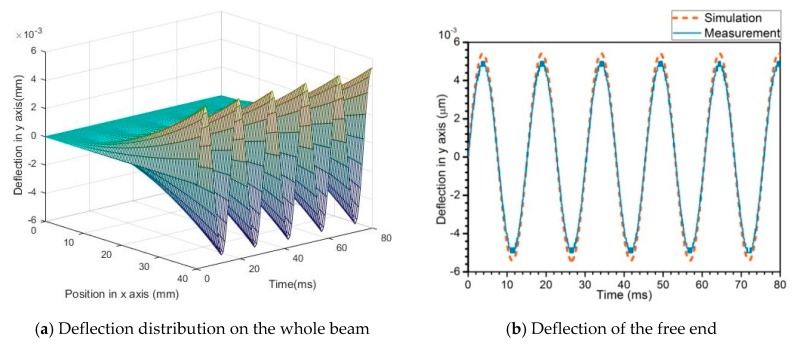
Deflection of the beam.

At the fundamental resonant frequency, the electrical responses including output voltage and power of the harvester connected with different pure resistance are tested. A sinusoidal base excitation is applied on the pedestal in this experiment. Figure 9a,b show the time responses of deflection on the whole beam and at the free end. The time response of deflection of any point on the beam is harmonic with resonance. The simulation value of the free end deflection is slightly larger than the measured value.

**Figure 10:**
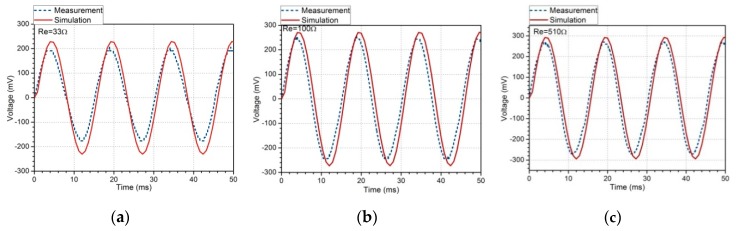
Comparison between numerical and experimental output voltage on external resistor. (**a**) 33 Ω external resistor, (**b**) 100 Ω external resistor, (**c**) 510 Ω external resistor.

**Figure 11:**
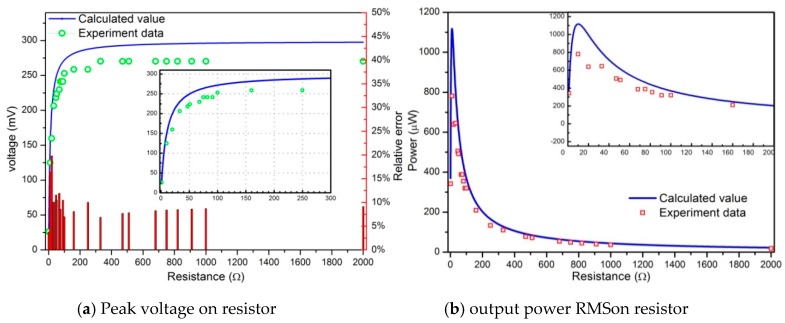
Output voltage and electrical power versus external resistive load at the fundamental resonance.

The harvesting prototype is next connected in series with external loads with different resistance, and the corresponding output voltage and electrical power on the resistor are shown in Figure 10 and Figure 11. The simulation value of voltage and power in theory are larger than the measurement value in the experiments. Most voltage errors range from 7% to 10%, as shown in the red bar chart in Figure 11a, which results in the error of power RMS shown in Figure 11b. The voltage calculation is not absolutely accurate, possibly due to the derivative involved in relations (18) and (21). Another reason is the inaccuracy of the simplified flux density estimated through the demagnetizing field and demagnetizing factor. This discrepancy may also result from the fact that the above model has not concerned the eddy current energy loss in the Fe-Ga alloy. In addition to the above possible reasons, the phenomenon may be also the result of a combination of factors acting together, including the field-dependent and stress-dependent elastic properties, stress saturation, non-constant magnetic permeability and so on. The voltage increases gradually with the increase of external resistance, and finally reaches a constant saturation state after 300 Ω (the experimental data and prediction value are about 270 mV and 297 mV, respectively, which is shown in the inset of Figure 11a). The maximum output power of 796 μW appears at the 10 Ω resistance, which is approximately equal to the impedance of the pick up coils (resistance 10 Ω, inductance 3.3 mH). The optimal volume power density (power divided by the total volume of the Iron gallium active layers) is about 2.653 mW/cm^3^, which is 9.5 times the power density of the Metglas 2605SA1-based magnetostrictive harvester (279 μW/cm^3^) designed by Hu and Xu [49], 2.4 times the magnetostrictive/piezoelectric composite harvester (1.1 mW/cm^3^) designed by Dai and Wen [50] and 5.4 times that of the electromagnetic energy harvester (495.5 μW/cm^3^) designed by Li and Yan [51]. Also, it can concluded that when Fe-Ga harvester serves as the power source for an electronics device whose impedance matches the harvester’s internal impedance, its output power performance is the best. However, the output voltage harvested by Fe-Ga is usually lower than the forward voltage drop of a diode, so it is not practical in practice, which may be solved by using a voltage booster.

**Figure 12:**
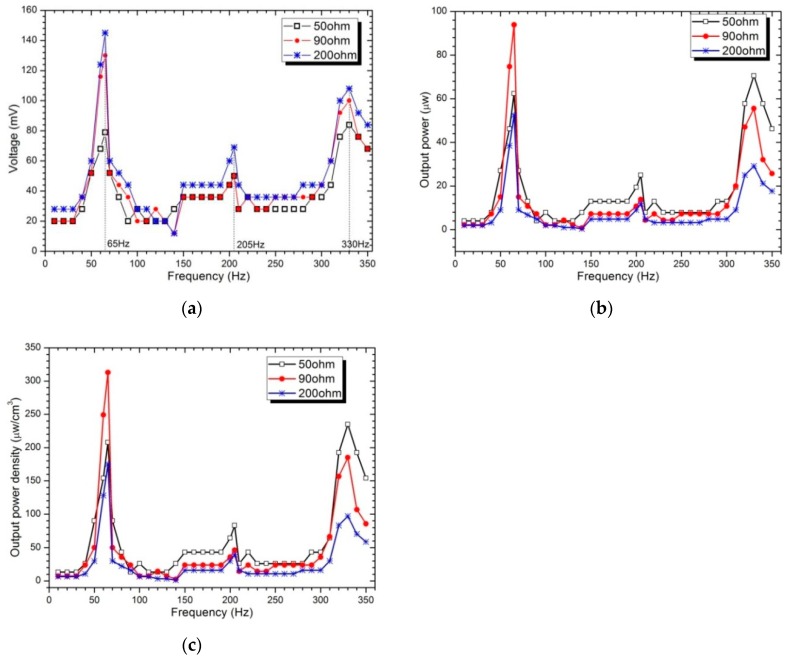
Voltage, power and power density versus excitation frequency below the third-order resonance frequency: (**a**) Voltage; (**b**) Power; (**c**) Power density. The harvester is connected with three different pure resistors: 50, 90 and 200 Ω; the vibration acceleration is 4 g.

Figure 12 depicts the output voltage, power and power density (frequency response curves within the first-three resonances) in terms of the excitation frequency for three different pure resistors (50, 90 and 200 Ω). The results show that once the excitation frequency coincides with one of the resonant frequencies, the harvested power, power density and output voltage increase accordingly. In addition, the maximum voltage and power are the largest in the first mode, and the maximum output voltage is about 145 mV, the maximum output power reaches 95 μw and the corresponding power density is 317 μw/cm^3^. With the increase of excitation frequency, the inductance impedance value increases, therefore, the power decreases at the second and third mode correspondingly. The harvested powers for 50 Ω and 90 Ω are considerably higher than that of higher load resistance 200 Ω. Unlike piezoelectric-based energy harvesters, the resonant frequencies of the Fe-Ga based harvester are considerably lower; as a result, in order to harvest the maximum possible energy under a base excitation with lower frequency, it is more appropriate to exploit Fe-Ga-based energy harvesters rather than piezoelectric harvesters. The optimal resistive load is normally a very small value, which is 10 Ω in the harvester involved in this paper shown in Figure 11b, compared to that of a piezoelectric harvester which is usually larger than 1 MΩ [52]. Thus, the Fe-Ga harvester is well suited to power electronic components whose resistive load is low.

### 4.4. Energy Harvesting Experiments under the Free Excitation

**Figure 13:**
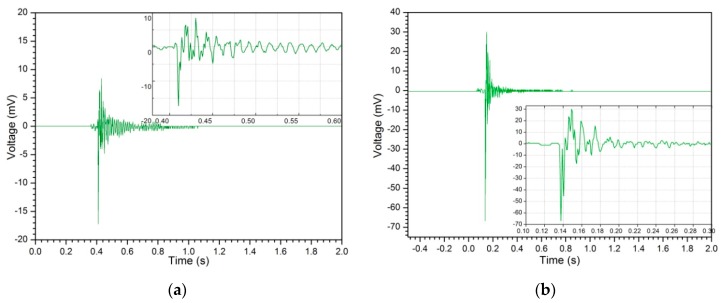
Voltage time-response at transient free vibration (0.010 Kg or0.050 Kg mass): (**a**) initial excitation is 0.098 N (0.010 Kg mass); (**b**) initial excitation is 0.49 N (0.050 Kg mass).

In the experiment, the transient excitation is exerted on the free end of the beam through suspending a weight via a rope, and then the free vibration of the beam is produced by cutting the rope. The harvester is connected in series to a 200 Ω resistance, and the generated voltage is measured by a digital oscilloscope with a high impedance probe. Figure 13 shows the voltage time-response to two sets of free excitations with different amplitude. The harvester also oscillates with resonance in free vibration as mechanical force is exerted on the free end and released suddenly. The resonance frequencies are all about 65 Hz for the two sets of excitations. Figure 13a shows the output voltage time-response of the harvester at a 0.098 N (0.01 Kg mass) initial excitation. The initial maximum voltage is about 17 mV. The input mechanical energy is 4.9 × 10^−5^ J and the total output electric energy is 0.55 × 10^−5^ J, and correspondingly the calculated energy harvesting and conversion efficiency is about 11.3%. Figure 13b shows the output voltage time-response at a 0.49 N (0.05 Kg mass) initial excitation. In this case, the initial maximum voltage rises to 67 mV. The input mechanical energy and output electric energy are 73.5 × 10^−5^ J and 7.2 × 10^−5^ J respectively. The energy harvesting and conversion efficiency is about 9.8%. The decay process from initial state to zero voltage lasted for approximately 0.66 s. This leads to the conclusion that the free vibration of the intermediate beam converts a low frequency periodic input force of several Hertz of order into resonance, which is about 65 Hz for the harvester designed in this paper. The output energy depends on the amplitude in the free vibration.

**Figure 14:**
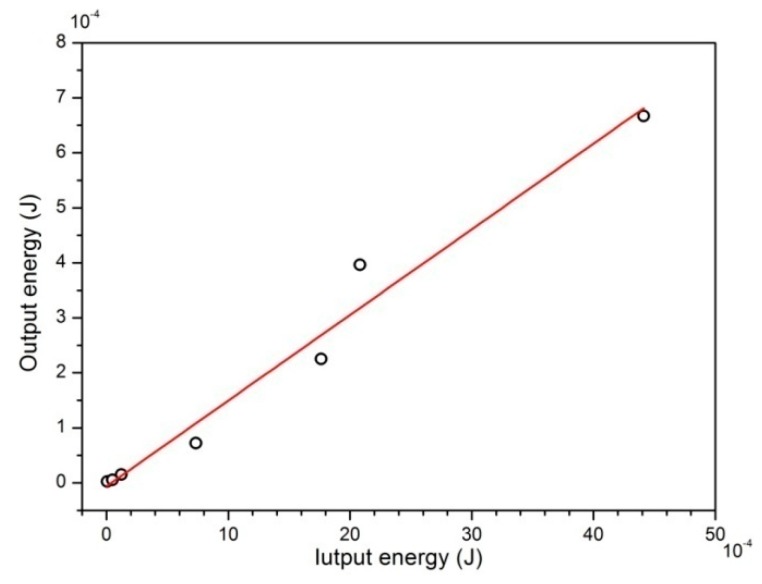
Output energy versus input energy.

Figure 14 shows the relationship between input energy and output energy under different initial excitations. It can be seen that there is an approximately linear relationship between input mechanical energy and harvested electrical energy. The results show that the slope of the curve is regarded as a constant, which means that the conversion efficiency is independent of free excitation amplitude. Energy harvesting and conversion efficiency corresponding to different initial excitation is shown in Table 2. The average conversion efficiency of the Fe-Ga harvester designed in this paper is 17.7%, which is higher than that of the magnetostrictive harvester with a parallel structure and tip mass (5.4% at 94 Hz resonance and 16% at 395 Hz resonance) designed by Ueno, Hu and Yamada [28].

### 4.5. The Effect of Pre-Magnetized Magnetic Field on Energy Harvesting

**Figure 15:**
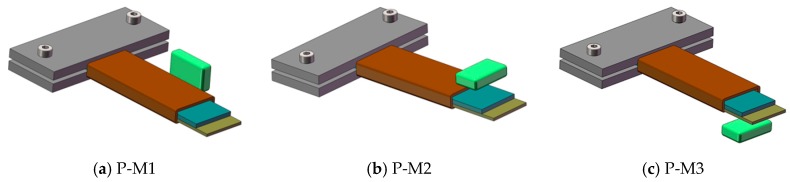
Position and layouts of the premagnetized magnet in the harvester. (**a**) magnet is on the right side of the beam (P-M1); (**b**) magnet is above the beam (P-M2); (**c**) magnet is arranged below the beam (P-M3).

**Figure 16:**
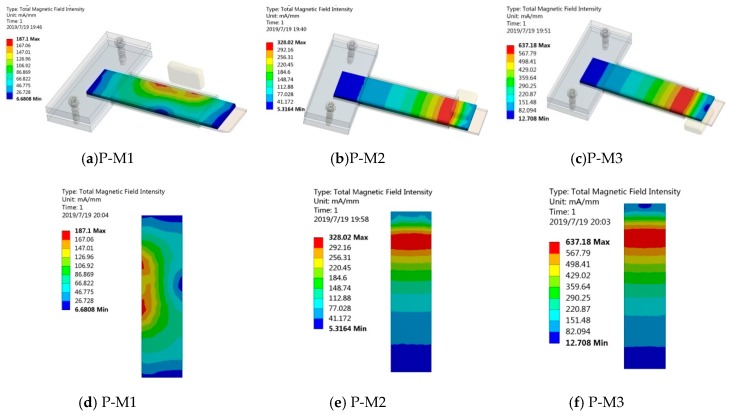
Magnetic field intensity calculation along the length of the Fe-Ga layer: (**a**), (**b**) and (**c**) are 3-D distribution; (**d**), (**e**) and (**f**) are 2-D distribution.

Some studies [53,54,55] have shown that pre-magnetized magnetic fields have an effect on the Villari effect of magnetostrictive materials, so we speculated that it also has an effect on the performance of Fe-Ga harvester. We adopt a permanent magnet to provide a pre-magnetized field to the Fe-Ga layer and select three different pre-magnetized positions and layouts to carry out this experiment. The positions and layouts of the premagnetized magnet in the harvester are shown in Figure 15. The layout where the magnet is arranged on the right side of the beam is represented by the symbol P-M1, when arranged above the beam free end it is represented by symbol P-M2, and when arranged above the beam free end it is represented by the symbol P-M3.In every case, four identical square permanent magnets of 20 mm × 9 mm × 2 mm are adopted. Firstly, the magnetic field intensity distributions of the harvesters with the above three kinds of magnet layouts are analyzed by the Workbench method. The finite element calculation results are shown in Figure 15 and Figure 16. The magnetic field intensity in the Fe-Ga layers magnetized by the P-M2 and P-M3 magnet layouts is distributed symmetrically along the width direction, which can ensure that the Fe-Ga layer is relatively fully utilized.

**Figure 17:**
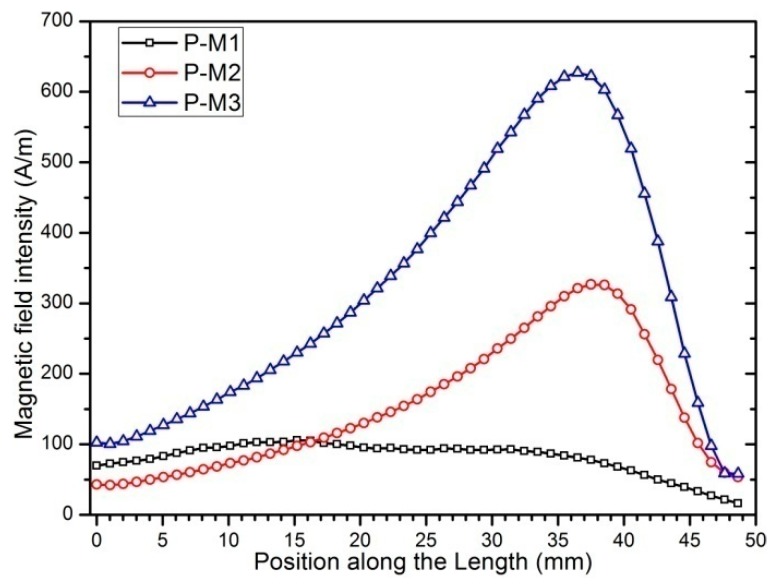
Comparison of the average magnetic field intensity along the length direction of the Fe-Ga layer with different pre-magnetized layouts.

From Figure 16 and Figure 17, it is noted that magnetic field intensity in Fe-Ga layer with the P-M3 layout is higher than that of the P-M2 and P-M1 ones, consequently, more magnetic domains will be stimulated. Correspondingly, the magnetic domain will rotate widely and expand greatly once it is excited by an external mechanical stress, and thus strongly modify the magnetic properties of Fe-Ga alloy due to the Villari effect. Therefore, it can be inferred that the Fe-Ga harvester with P-M3 layout may have better performance.

**Figure 18:**
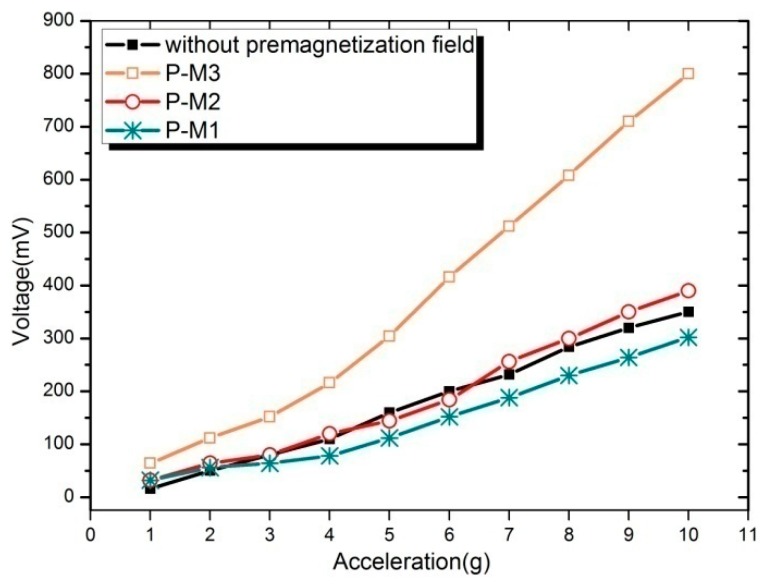
Voltage of the harvester without and with pre-magnetized magnet.

Figure 18 shows the output voltage of the harvester without and with pre-magnetized magnet. The experiments are conducted under a base excitation of 65 Hz. It can be seen that voltage increases with the excitation acceleration. However, different magnet positions lead to great differences in the voltage. When the magnet is placed below the free end of Fe-Ga beam (P-M3), the voltage rises rapidly with the increasing of acceleration and the voltage amplitude reaches to 800 mV, which is more than twice as much as that without a pre-magnetized magnet (350 mV).

**Figure 19:**
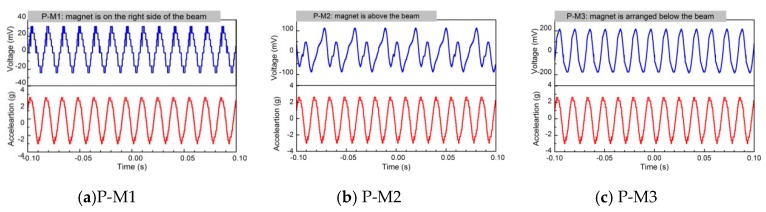
Open-circuit output voltage waves of the harvesting prototype with different layout of premagnetized magnet monitored by digital oscilloscope: (**a**) P-M1; (**b**) P-M2; (**c**) P-M3.

Correspondingly, the output power will also much higher than that of without pre-magnetized magnet. Similarly, the energy conversion efficiency will be higher by 17.7%. On the other hand, it is also observed that not all the pre-magnetization fields can improve the energy harvesting ability in all cases. For example, not all the voltage generated by the harvester with pre-magnetized magnet on the above of free end of Fe-Ga beam (P-M2) or parallel to the length direction (P-M1) are larger than that of without magnet all the times. This indicates that the pre-magnetization field has an impact on Fe-Ga harvester’s harvesting capability; moreover, the results are related to the location of magnet. From Figure 19, the magnet with P-M3 layout is relatively optimal, which is consistent with the above theoretical speculation because the voltage wave shape is better than the others, and its amplitude is the highest too. Obtaining more details about the pre-magnetization field effect requires a detailed modeling of the whole structure, which will be solved in our future research. In the future, we will also study the working performance of the harvester pre-magnetized and create an optimal design.

### 4.6. Experiments of Harvester Applied to Power LEDs and Digital Display Tubes

**Figure 20:**
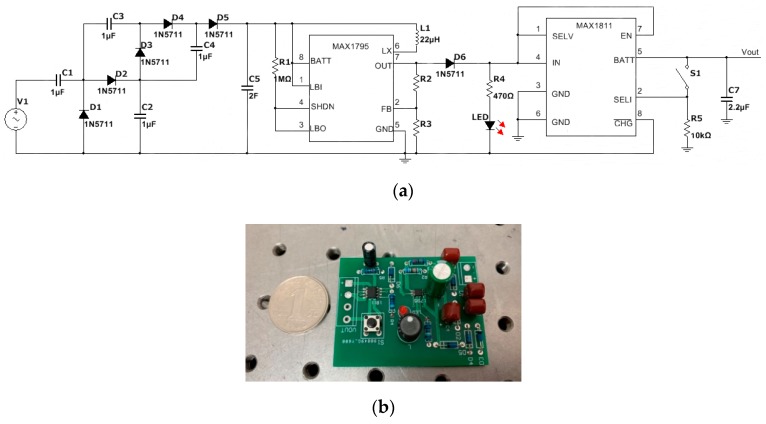
Schematic diagram (**a**) and actual printed circuit board (**b**) of energy harvesting and management circuit.

The energy storage circuit designed for the proposed Fe-Ga harvester consists of three parts: a voltage quadruple rectifier circuit for rectifying and charging, a super capacitor for energy storage, and an intelligent voltage regulator. Compared with chemical rechargeable batteries, supercapacitors exhibit excellent performances, including lower cost, instant charging, about 10 times the charging and discharging efficiency, semi-permanent life cycle, smaller size (1/7 of AA batteries). The output voltage of the harvester is AC and less than 1 V. Consequently, on the one hand, it is not enough to charge the supercapacitor, so it needs to be boosted. On the other hand, AC needs to be rectified into DC due to the fact electronic components usually work in DC mode. Although half-wave or full-wave rectifier bridges can convert AC to DC, the DC voltage is even lower due to the forward voltage drop of the diode. In order to overcome this obstacle, we adopt a voltage quadruple rectifier circuit as the rectifier and boost circuit, which is able to realize AC-DC rectification, filtering and raising DC voltage level concurrently. An intelligent regulator is the logic unit in the energy harvesting module. It can clamp the output DC voltage level of the super- capacitor, optimize the charging performance and self-turn on and off. Here, we choose an efficient MAX1795 DC-DC boost converter, which has characteristics, such as circuit self-shutdown, completely switching off the input and output, improved efficiency and it avoids the energy consumption of peripheral components. The MAX1795 consumes only 25 μA static current and 2 μA turn-off current. By setting the peripheral circuit shown in Figure 20a, the output voltage of MAX1795 can be adjusted through adjusting the resistor R_4_ according to the requirement of external load. The schematic diagram and the actual printed circuit board of the energy harvesting and management circuit are shown in Figure 20.

**Figure 21:**
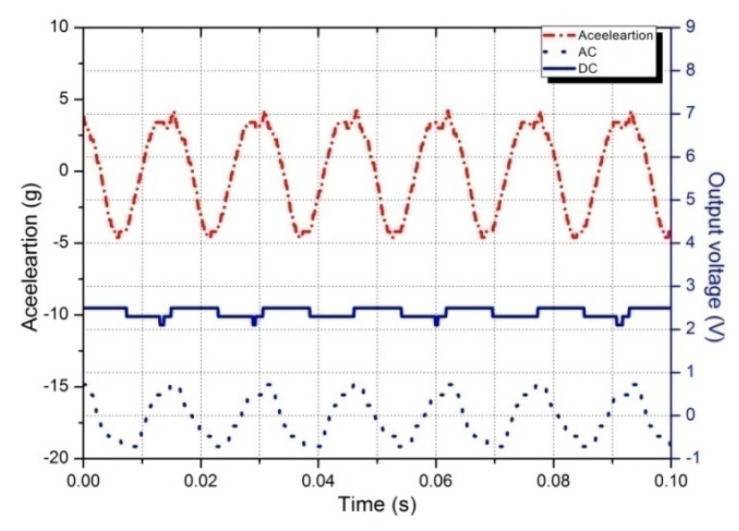
AC output from the harvester and DC output from the voltage quadruple rectifier circuit.

Figure 21 shows the measured vibration acceleration, AC voltage of the harvester and DC output of the voltage quadruple rectifier circuit. When a 4 g sinusoidal vibration is exerted on the harvester under the fundamental resonant frequency, it outputs 0.72 V AC voltage, which is monitored by a digital oscilloscope and expressed by dotted lines. Theoretically, the DC output of the voltage quadruple rectifier should be 2.88 V. However, the actual DC voltage is about 2.45 V due to the forward voltage drop of diode, which is expressed by a solid line.

**Figure 22:**
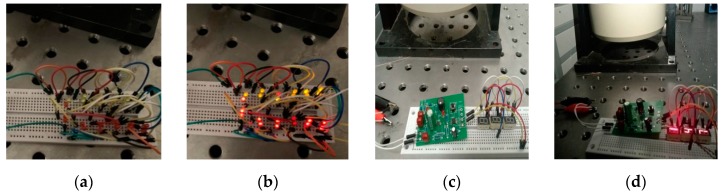
Photograph of a verification experiment for power generation effect: in (**a**) and (**c**), harvester was not working; in (**b**) and (**d**), harvester was working and multi-LEDs and digital display tubes were lighted up.

Finally, we use the rectified DC output to power twenty-four parallel-connected LEDs, including fourteen red and ten yellow, or three digital display tubes. As shown in Figure 22, twenty-four LEDs and three digital display tubes are kept lit up by the prototype. It may be the first time a Fe-Ga alloy vibration harvester can keep multi-digital display tubes lit up.

This test validates that the prototype has an excellent power generation capability and robustness, and it suggests some potential applications for the Fe-Ga alloy magnetostrictive vibration harvester such as LED indicating devices and so on. In the future, we will further study the working characteristics of its charging, storage and discharge processes and we will try to apply the harvester to the power supply of wireless sensor nodes.

## 5. Conclusions

A Fe-Ga-based cantilever vibration harvester utilizing the coupling characteristics between the Villari effect and the Faraday electromagnetic induction was proposed. It was a composite cantilever beam composed of a metal base layer and a Fe-Ga alloy layer, surrounded by a pick up coil. The harvester was capable of harvesting base excitations with low or high frequency, and free excitations with very low frequency. The energy harvesting performance of the harvester prototype, including its resonance characteristics, open-circuit output voltage-frequency response, voltage vs. vibration amplitude characteristic under base excitation, influence of external resistance, energy harvesting performance under free excitation, the influence of pre-magnetization and so on was studied systematically and carefully by experiments. The fundamental natural frequency is about 65 Hz, and the energy harvesting capacity is the strongest in this situation. The output voltage increases gradually with the increase of external resistance, and finally reaches a constant saturation state. The maximum output power appears at a 10 Ω resistance, which is approximately equal to the impedance of the pick up coil. In terms of the volume power density, the harvester without pre-magnetized magnet when in series with the optimal resistor load displays a value of 2.653 mW/cm^3^ and when it is in series with a 200 Ω resistance(not the optimum matching impedance), its average conversion efficiency is about 17.7%. A pre-magnetization field has an impact on Fe-Ga harvester’s harvesting capability, moreover, the results are related to the location of the magnet. In this paper, a magnet location below the free end of the Fe-Ga beam is optimal. The output voltage of the harvester is rectified, boosted and stored by an energy harvesting and management circuit, and the sustainable power generation capacity of the fabricated prototype is validated through keeping multi-LEDs and digital display tubes lit up.

## Figures and Tables

**Table 1:** Main parameters.

Symbol	Description	Value
λ_1_	nontrivial solution of parameter λ of natural frequency (first order)	1.875
λ_2_	nontrivial solution of parameter λ of natural frequency (second order)	4.694
λ_3_	nontrivial solution of the parameter λ of natural frequency (third order)	7.885
λ_4_	nontrivial solution of the parameter λ of natural frequency (forth order)	10.996
A_s1_	constant coefficient of the mode shape function (first order)	0.734
A_s2_	constant coefficient of the mode shape function (second order)	1.019
A_s3_	constant coefficient of the mode shape function (third order)	0.999
A_s4_	constant coefficient of the mode shape function (forth order)	1.00003
*E*_s_	elastic modulus of basal layer of Fe-Ga layer	70 GPa
*E*_G_	elastic modulus of basal layer	128 GPa
*E*_c_	elastic modulus of pick up coil layer	0.45 GPa [47]
*m*	the mass per unit length of the beam	0.35 × 10^−3^ Kg/mm
*l*	Effective cantilever beam length	40 mm
κ	irreversible loss coefficient	0.2
γ	coefficient of anhysteretic magnetization	4.2 × 10^3^ A/m
M_s_	saturation magnetization	6 × 10^4^ A/m
λ_s_	saturation magnetostriction coefficient	160 ppm
μ_0_	vacuum permeability	4π × 10^−7^ A/m
τ	magnetic coefficient of external molecule	0.1 × 10^−3^
ξ	energy coupling parameter per unit volume	300 A/m

**Table 2:** Energy harvesting and conversion efficiency.

Input Mechanical Energy (10^−4^)	Output Electrical Power (10^−4^ J)	Conversion Efficiency
0.06	0.026	43.1%
0.49	0.055	11.3%
1.23	0.153	12.5%
7.35	0.721	9.8%
17.6	2.251	12.8%
20.8	3.966	19%
44.1	6.668	15.1%
